# The effect of nurse‐led Internet‐based cognitive behavioural therapy for insomnia on patients with cardiovascular disease: A randomized controlled trial with 6‐month follow‐up

**DOI:** 10.1002/nop2.817

**Published:** 2021-02-20

**Authors:** Sandra Siebmanns, Peter Johansson, Martin Ulander, Linda Johansson, Gerhard Andersson, Anders Broström

**Affiliations:** ^1^ Department of Nursing Science School of Health and Welfare Jönköping University Jönköping Sweden; ^2^ Department of Health, Medicine and Care Linköping University Norrköping Sweden; ^3^ Department of Internal Medicine and Department of Health, Medicine and Care Linköping University Norrköping Sweden; ^4^ Department of Clinical Neurophysiology Linköping University Hospital Linköping Sweden; ^5^ Institute of Gerontology Aging Research Network‐Jönköping School of Health and Welfare Jönköping University Jönköping Sweden; ^6^ Department of Behavioral Sciences and Learning Linköping University Linköping Sweden; ^7^ Department of Clinical Neuroscience Karolinska Institutet Stockholm Sweden

**Keywords:** cardiovascular disease, cognitive behavioural therapy, insomnia, internet‐based, nurse support

## Abstract

**Aim:**

To test the effect of nurse‐led Internet‐based cognitive behavioural therapy for insomnia (I‐CBTI), tailored for patients with cardiovascular disease (CVD), with a 6‐month follow‐up.

**Design:**

A two‐arm parallel‐group randomized controlled trial (RCT) registered at clinicaltrials.gov (NTC03938805) and reported according to the CONSORT checklist.

**Methods:**

Forty‐eight patients (mean age 72 years, 65% men) diagnosed with CVD and insomnia were randomized to either 9‐week nurse‐led I‐CBTI with support, or an Internet‐based self‐study programme without support (control group). Insomnia Severity Index (ISI) and Short Form Health Survey (SF‐12) were used as primary and secondary outcomes.

**Results:**

ISI showed a significant treatment effect of I‐CBTI compared to the control group at 9‐week follow‐up. The mean ISI score in the I‐CBTI group at 9 weeks post‐treatment was maintained at the 6‐month follow‐up. Patients' adherence to I‐CBTI was associated with a better effect on both the ISI and SF‐12.

## 1 INTRODUCTION

Insomnia is a frequent problem among patients with cardiovascular disease (CVD), with prevalence as follows in patients with CVD (Javaheri & Redline, 2017) – 44% (Taylor et al., 2007), acute coronary disease 37% (Coryell et al., 2013), heart failure 50% (Redeker et al., 2010), versus 10% in the general population (Grandner et al., 2016). Insomnia, together with cardiac symptoms, can aggravate physical, cognitive and emotional symptoms (i.e. depression, anxiety, fatigue and pain) and cause poor quality of life (Conley & Redeker, 2015; Da Costa et al., 2017; Johansson et al., 2011). Although the combination of CVD and insomnia is associated with poor prognosis (i.e. increased morbidity and mortality) (Conden & Rosenblad, 2016; Javaheri & Redline, 2017; Sofi et al., 2014), the health care often fails to address insomnia or to offer sufficient insomnia treatment for this patient group (Conley & Redeker, 2015). According to the ICSD‐3 criteria, insomnia is defined as difficulty initiating or maintaining sleep, or waking up earlier than desired, at least three times a week for a minimum of 3 months and with at least one reported daytime impairment related to the night‐time sleep difficulty (Riemann et al., 2017). The associations between insomnia and CVD are thought to be caused at least in part by biological processes (i.e. by an increased level of cortisol) (Grandner et al., 2016), leading to hyperarousal, reduced sleep duration and increased systemic inflammation that causes enhanced levels of atherogenesis, lipids and insulin resistance, which work as mediators to develop CVD (Javaheri & Redline, 2017). On the other hand, poor quality of life, fatigue and functional impairment associated with cardiac symptoms and/or sleep deprivation are common among patients with CVD (Conley & Redeker, 2015), which can further worsen sleep and the prognosis. Evidence suggests that cognitive behavioural therapy for insomnia (CBT‐I) can improve insomnia and CVD outcomes, and can possibly increase quality of life (Conley & Redeker, 2015).

## 2 BACKGROUND

Research on CBT‐I in both general (Buenaver et al., 2019) and comorbid populations (Geiger‐Brown et al., 2015) (i.e. psychiatric disorders, chronic pain, cancer and depression) has shown positive outcomes on insomnia but the treatment is underused in clinical practice due to a shortage of CBT therapists (Geiger‐Brown et al., 2015). A suitable alternative is therefore Internet‐based CBT for insomnia (I‐CBTI), which has been found to be as effective as face‐to‐face treatment when used in the general population (Blom et al., 2015). Earlier Internet‐based CBT studies addressing different health conditions, where some have been nurse‐led I‐CBT, have shown positive treatment effects (Johansson et al., 2019; Lundgren et al., 2016; Mehta et al., 2019), making this a possible approach for nurses when caring for patients with CVD and insomnia.

The recent European guidelines for the diagnosis and treatment of insomnia recommend training other healthcare professionals (i.e. registered nurses) to increase access to insomnia treatment (Riemann et al., 2017). Earlier nurse‐led I‐CBT studies to treat depression in patients with heart failure (Johansson et al., 2019) and CVD (Lundgren et al., 2016), as well as technician‐supported studies on depression, have shown positive effects when the deliverers took brief training courses and had access to guideline scripts that covered topics in the programmes (Titov et al., 2010).

Regarding content, Heenan et al. (2020) showed in a pre–post I‐CBTI intervention for patients with CVD that cardiac‐relevant components in the intervention can lead to significant improvement in insomnia and lower levels of anxiety and depression. To include CVD‐specific elements (i.e. psychoeducation about CVD) in an I‐CBTI intervention could therefore be beneficial, to improve both insomnia and cardiac health. There is to our knowledge no published randomized controlled trial (RCT) with long‐term follow‐up that has used a tailored I‐CBTI intervention for patients with CVD and insomnia supported by a nurse. A nurse‐led I‐CBTI intervention tailored for patients with CVD is an important step forward for increasing treatment access and facilitating improvement in sleep and cardiac health. Therefore, the aim of this RCT was to test the effect of a nurse‐led I‐CBTI programme, with a 6‐month follow‐up, for patients with CVD and insomnia.

## 3 METHOD

### 3.1 Study design and sample

A parallel RCT with a 6‐month follow‐up was used and reported according to the CONSORT checklist (Schulz et al., 2011). Participants were recruited via registers of diagnosis from six primary care centres in southern Sweden. The search in the diagnosis registers included diagnostic codes from the International Classification of Disease (ICD‐10) and/or the Classification of Diseases and Health Problems 1997 (KSH97‐P, a simplified version of ICD‐10, used by the Swedish primary care system). Inclusion criteria were patients ≥ 18 years old, verified diagnosis of insomnia, verified CVD diagnosis, that is: angina pectoris (I20.9, I20.9P), myocardial infarction (I25.2, I25.‐P), heart failure (I50.9, I50.‐), atrial fibrillation and atrial flutter (I48.9, I48.‐), and arrhythmia NOS (I49.9, I49). The CVD diagnosis had to be registered during at least two different healthcare visits to minimize the risk of including patients misdiagnosed with CVD. Exclusion criteria were no access to the Internet, computer or smartphone, the need for an interpreter to understand Swedish text and language, being engaged in shift work, cognitive disabilities, severe psychiatric disease, severe somatic disease, comorbid untreated sleep disorders (i.e. sleep apnoea, severe restless leg syndrome or circadian rhythm disorders), epilepsy, drug abuse or expected survival less than 12 months.

### 3.2 Data collection

**FIGURE 1:**
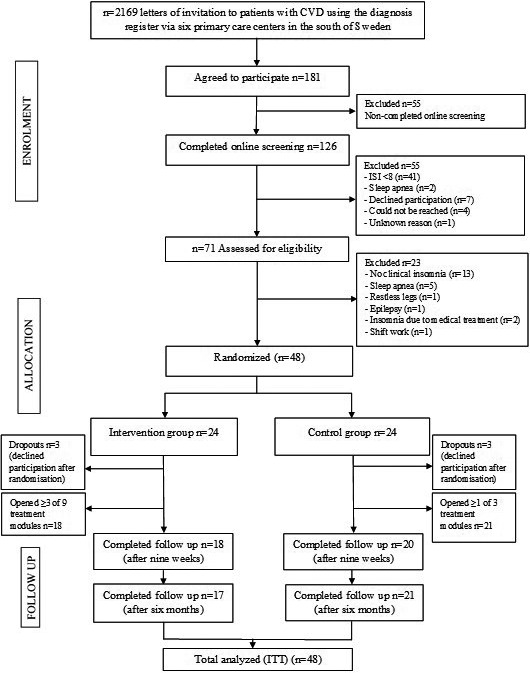
Flow chart (CONSORT) of the inclusion process and study compliance

Letters with information about the study were sent out to 2,169 patients (Figure 1). Patients who returned written informed consent to participate (*N* = 181) were emailed a link to complete a web‐based questionnaire (on demography, sleep quality and physical and mental health). A total of 126 participants completed the web‐based screening. Participants that reported at least subthreshold insomnia symptoms according to the Insomnia Severity Index (*N* = 85) were contacted by telephone and interviewed by a registered nurse to check for inclusion and exclusion criteria. The participants were then scheduled to meet with a physician specializing in sleep, who diagnosed insomnia according to the ICSD‐3 criteria (Riemann et al., 2017). Reasons for exclusion after the web‐based screening, the nurse interview or meeting with the physician are presented in Figure 1. Participants eligible for randomization (*N* = 48) then met a registered nurse to receive further information about the study plan.

### 3.3 Randomization process

Participants were consecutively randomized with a block size of two (generated by an independent statistician using Stata version 13 proc Ralloc StataCorp LLC) to either the I‐CBTI intervention or the control group. The participants were blinded to the randomization assignment until their first login to the Iterapi platform. A total of 48 participants were randomized to I‐CBTI (*N* = 24) or the control group (*N* = 24).

### 3.4 Measures

Data regarding the following measures were collected at the same time points for both the intervention and control groups.

#### 3.4.1 Insomnia symptoms (Primary outcome)

The Insomnia Severity Index (ISI) (i.e. data collected at baseline, 9 weeks and 6 months) measures the participants' perceived severity of insomnia symptoms, such as difficulties falling asleep, staying asleep, number of awakenings, early awakening and sleep satisfaction. The scale includes seven items, scored on a 0–4 scale, that are summed to a range of 0–28. The score can be divided into four categories: no clinical insomnia (0–7), subthreshold insomnia (8–14), clinical insomnia of moderate severity (15–21) and severe clinical insomnia (22–28) (Sarsour et al., 2010). The ISI has been tested and validated for its reliability (Morin et al., 2011; Thorndike et al., 2011). Cronbach's *α* (i.e. internal consistency) for the ISI at baseline was 0.68.

#### 3.4.2 Quality of life (Secondary outcome)

The Short Form Health Survey 12 (SF‐12) was used to measure the participants' quality of life (i.e. data collected at baseline and 6 months). The 12 items rated on a five‐point Likert scale cover eight concepts: physical functioning, role physical, bodily pain, general health, vitality, social functioning, role emotional and mental health. Two reliable and valid summary scores are reported—a physical component score (PCS) and a mental component score (MCS). The SF‐12 has been tested and validated for its reliability (Muller‐Nordhorn et al., 2004). Cronbach's α for SF‐12 at baseline was 0.82.

#### 3.4.3 Adherence to I‐CBTI (Secondary outcome)

Participants in the intervention group were labelled as adherent or non‐adherent after a consensus agreement of members in the group (i.e. including researchers with extensive experience on ICBT in other diagnostic groups) based on visual inspection of the data regarding logins, completion of assignments and completed modules, as well as in relation to adherence levels used in other ICBT studies (Blom et al., 2015; Lancee et al., 2016). A value of ≥5 completed modules was defined as adherence to the I‐CBTI intervention.

### 3.5 Design of the intervention

When randomized, the participants received an email via an encrypted Iterapi platform for Internet‐based psychological treatment (Vlaescu et al., 2016) and were given an individual login (username and password), and a request to change the password after the first login. The 9‐week I‐CBTI programme included nine modules (Table 1) with support from a registered nurse. The control group underwent a self‐study programme that contained three modules (the same content as the I‐CBTI intervention's first three modules) without the ability to receive support. For ethical reasons, the self‐study program was developed to include information that could be considered useful by the participants not randomized to the I‐CBTI intervention. The participants in the control group had access to the three modules for the same period that the intervention group had access to the I‐CBTI program. In the I‐CBTI intervention, module one was an introduction to the program. Module 2 was a collection of three modules describing three different CVD conditions (i.e. ischaemic heart disease, heart failure and atrial fibrillation and flutter), designed by four nurses and a physician with extensive experience of care for patients with CVD. Module 2 was delivered to the participants with reference to their CVD diagnosis and was designed to inform and educate in pathophysiology, treatment alternatives and self‐care recommendations of CVD. Module 3 included information about sleep and sleep hygiene, and module 4 had information about insomnia and its relation to CVD. Modules 5–8 included psychoeducation to improve sleep as well as behavioural interventions (i.e. stimulus control, sleep restriction, relaxation, management of stress and dysfunctional thoughts about sleep). To minimize the adverse daytime effects of sleep restriction (i.e. fatigue, extreme sleepiness, reduced motivation, irritability and changes in appetite) (Kyle et al., 2011), the participants were initially recommended to perform sleep restriction once or twice a week and then gradually increase the use of sleep restriction each week. Module 9 was a summary of the intervention, and the participants were asked to assess what they had learned and how they would handle setbacks in the future. The text in module 1 and text and quizzes in modules 3–9 were developed by researchers and clinicians (i.e. nurses, physicians and psychologists) with extensive experience of CVD care and sleep medicine. In total, there were 20 assignments in the I‐CBTI intervention, with one to four assignments per module. Every assignment was specific to each module (Table 1), and all modules ended with a quiz on the module's topic. Moreover, all modules in the I‐CBTI programme included a short pre‐recorded video in which the supporting nurse gave a brief description of the content. The support was delivered by one registered nurse, trained in I‐CBT support, who if needed had access to written I‐CBT guidelines, as well as support from a psychologist, a sleep physician and two nurses specializing in sleep and CVD. When participants completed assignments, they documented the results and saved them, which made it possible for the supporting nurse to give written feedback (i.e. confirming, encouragement and reflection). If the participants did not complete the assignments by the end of the week, they received one or two reminders. The feedback was given by the nurse within 24 hr, mainly on work weekdays in office hours, and was adapted to the participants' needs, depending on the size, complexity and number of completed assignments. All communications were carried out in a secure encrypted mail‐system within the Iterapi platform (Vlaescu et al., 2016). All participants could log into the programmes (i.e. there was the same availability for both the intervention and control groups) within 2 months of completion and were able to download the content of the modules to a PDF to read or save for future use. The I‐CBTI intervention and self‐study program were developed to be suited for users with various levels of computer literacy.

**TABLE 1:** Overview of the I‐CBTI (intervention group) and the self‐study program (control group)

Module	I‐CBTI and self‐study program module^a^	Aim	Assignments in the I‐CBTI program
1	Introduction^a^	To inform and create a commitment for the program	Establish expectations and goals for the program (one assignment)
2	Living with cardiac disease^a^	To impart knowledge of what cardiac disease (ischaemic heart disease, heart failure and atrial defibrillation/flutter) is, treatments and what it is like to live with a cardiac disease	Identify situations when heart symptoms appear and give suggestions for change. (Two assignments plus a quiz)
3	Sleep^a^	To impart knowledge about sleep and how it is measured, our need for sleep and the factors affecting sleep	To fill out a sleep diary. (One assignment plus a quiz)
4	Heart disease and sleep problems	To impart knowledge about what will happen when sleep is insufficient, and information about sleep medicine	To fill out a sleep diary. (One assignment plus a quiz)
5	Stimulus control	To initiate the use of stimulus control to reduce the time spent awake in bed	To initiate stimulus control and document the results. (One assignment plus a quiz)
6	Sleep restriction	To initiate the use of sleep restriction to limit the time spent in bed	To plan and initiate sleep restriction and document the results. (One assignment plus a quiz)
7	Thoughts that contribute to sleeping badly	To impart knowledge about how thoughts and perceptions can have negative effects on sleep	To identify thoughts regarding sleep and describe the perceptions of the thoughts. (One assignment plus a quiz)
8	Stress related to heart disease that can contribute to sleep problems	To impart knowledge about the effects of stress and how to reduce those effects on sleep	To try relaxation and identify helpful and unhelpful thoughts connected to sleep. (One assignment plus a quiz)
9	Completion	To evaluate new knowledge and changes made through the program and make plans to maintain these changes to improve sleep	To fill out a sleep diary and evaluate new knowledge and changes made and how setbacks can be managed. (Three assignments and a quiz)

Note

The participants in the control group had access to the three modules during the same time duration as the intervention.

^a^ Self‐study program includes modules 1–3 (i.e. the same content as the I‐CBTI intervention's first three modules), but without the assignments. No support was delivered by the nurse to the members in the control group.

### 3.6 Sample size calculation

The sample size calculation was based on screening > 2,000 primary care patients diagnosed with CVD. The prevalence of insomnia in patients with various CVD diagnoses differs depending on the diagnosis but could be estimated to vary between 25%–45% (Conley & Redeker, 2015; Da Costa et al., 2017; Johansson et al., 2011). Considering a varied percentage of self‐perceived sleep problems, the current study's exclusion criteria and an acceptance rate of approximatively 35%, around 200 patients were likely to be available for participation in the web‐based questionnaire. Out of the individuals who responded correctly to the web‐based questionnaire and reported insomnia causing daytime impairment, approximatively 60% could potentially be excluded during the clinical examination due to lack of a verified insomnia diagnosis (Riemann et al., 2017), the presence of sleep apnoea or other sleep disorders (Jackson et al., 2015), or presence of exclusion criteria. Therefore, approximatively 60 patients were likely to be available for randomization in the study. Based on a medium size effect of 0.5 and *α* = 0.01 (*Z* = 1.96), and Power of 0.80 (*Z* −0.84), 60 participants (30/group) were required.

### 3.7 Data analysis

IBM SPSS statistics data editor version 26 was used for the analyses. The comparison of participants' demographic data between groups was carried out using descriptive statistics. Fisher's exact test for dichotomous variables and an independent sample *t* test for normally distributed variables (age, mean ISI and SF‐12) were used. The primary analysis was based on intention to treat, using Expectation‐Maximization (EM), an imputation approach that estimates unmeasured data iterated in two alternating steps (Blankers et al., 2010). EM is a compatible imputation method when using general linear regression. The missing data at follow‐up (9 weeks and 6 months) were 20.8%, and the MCAR (Missing Completely at Random) test for the missing data was non‐significant (Little's MCAR test: Chi‐Square = 0.000, *df* = 146, Sig. = 1.000). Demographic variables that correlated with primary and secondary outcomes were used as predictors when EM was carried out. A general linear regression (ANCOVA) was used to test the mean between‐group treatment differences between 9 weeks and 6 months, adjusted for baseline values as covariates with a significance level of 0.05 (95% CI). Effect size was calculated using Cohen's *d* on both the imputed and unimputed outcome data (*d* = M1 − M2/SD1 + SD2), where 0.20 is considered a small effect, 0.50 medium and 0.80 is large (Field, 2018). Post hoc analyses on primary and secondary outcomes were carried out for the I‐CBTI group based on adherence using Wilcoxon signed ranked test due to low number of cases. The between‐group effect of adherence in the non‐adherent group was not possible due to the small sample size.

### 3.8 Ethics

The research project was carried out in accordance with international ethical guidelines for health‐related research involving humans, and the study was approved by the Regional Ethical Council, Linköping University (Dnr 2015/258‐31, 2017/378‐32). The trial is registered at clinicaltrials.gov (NTC03938805).

## 4 RESULTS

### 4.1 Baseline characteristics

The baseline characteristics of the participants are presented in Table 2. There were no significant differences between the groups at baseline.

**TABLE 2:** Baseline characteristics

	Total (*N* = 48)	ICBT group (*N* = 24)	Control group (*N* = 24)
Age, *M* (*SD*)	72.52 (9.81)	72.46 (9.45)	72.58 (10.37)
Gender (%)			
Male	31 (64.6)	17 (70.8)	14 (58.3)
Female	17 (35.4)	7 (29.2)	10 (41.7)
Living situation (%)			
Single	14 (29.2)	7 (50)	7 (50)
Cohabitation (partner/children/other)	34 (70.8)	17 (70.8)	17 (70.8)
Educational level (%)			
Elementary school (7–9 years)	9 (18.8)	3 (12.5)	6 (25)
Senior high school (11–13 years)	15 (31.3)	8 (33.3)	7 (29.2)
University	24 (50)	13 (54.2)	11 (45.8)
Cardiovascular disease (CVD) (%)^a^			
Myocardial infarction	25 (52.1)	11 (45.8)	14 (58.3)
Angina pectoris	14 (29.2)	4 (16.7)	10 (41.7)
Heart failure	3 (6.3)	2 (8.3)	1 (4.2)
Atrial fibrillation or flutter	10 (20.8)	4 (16.7)	6 (25.0)
Arrhythmia NOS	9 (18.8)	4 (16.7)	5 (20.8)
Comorbidities (%)			
Hypertension	14 (29.2)	7 (29.2)	7 (29.2)
Diabetes	7 (14.6)	4 (16.7)	3 (12.5)
Dyslipidaemia	21 (43.8)	12 (50)	9 (37.5)
Lung disease	5 (10.4)	2 (8.3)	3 (12.5)
Total number of diagnosis Median, (*SD*)	2 (1.003)	2 (1.022)	2 (0.989)
Body‐mass‐Index (BMI) (%)			
BMI < 25	17 (35.4)	10 (41,7)	7 (29.2)
BMI > 25	31 (64.6)	14 (58.3)	17 (70.8)
Pharmacological CVD treatment (%)			
RAAS	28 (58.3)	13 (54.2)	15 (62.5)
Beta blockers	27 (56.3)	14 (58,3)	13 (54.2)
Anticoagulants	34 (70.8)	17 (70.8)	17 (70.8)
Statins	27 (56.3)	13 (54,2)	14 (58.3)
Diuretics	3 (6.3)	0 (0.0)	3 (12.5)
Vasodilators	3 (6.3)	0 (0.0)	3 (12.5)
Rhythm stabilization agents	9 (18.8)	4 (16.7)	5 (20.8)
Total number of CVD‐medication (MD) (Range)	3 (6)	3 (5)	3 (6)
Sleep medication	19 (39.6)	9 (37.5)	10 (41.7)
Insomnia Severity Index (ISI), *M* (*SD*)			
ISI score baseline	16.10 (3.959)	15.92 (3.878)	16.29 (4.112)
SF‐12 baseline score, M			
Physical component score	40.09 (9,09)	40.46 (8.27)	39.71 (10.00)
Mental component score	48.19 (11.82)	50.07 (11.68)	46.30 (11.89)

Note

There were no significant differences between the groups at baseline.

^a^ 11 participants had two or more CVD diagnosis.

### 4.2 Primary outcomes

The intention to treat analysis of ISI showed a moderate, significant treatment effect of the I‐CBTI compared to the control group (−2.9 between‐group mean difference, 95% CI −4.9 to −0.9, *p* = .004, Cohen's *d* = 0.73) at 9 weeks (Table 3). The improvement in ISI score in the I‐CBTI group at the post‐treatment follow‐up was maintained at the 6‐month follow‐up (11.64 vs. 11.03, *p* = .943). However, the between‐group effect at 6 months was not significant (−2.1 between‐group mean difference, 95% CI −4.7 to 0.5, *p* = .111, Cohen's *d* = 0.47). Unimputed data for insomnia (Appendix 1) showed a significant between‐group mean difference for the ISI score from baseline to 9 weeks (−3.3 between‐group mean difference, 95% CI −5.7 to −0.8, *p* = .004, Cohen's *d* = 0.72), but not at 6 months (−2.6 between‐group mean difference, 95% CI −5.9 to 0.8, *p* = .127, Cohens *d* = 0.79).

**TABLE 3:** Treatment effect on primary and secondary outcomes from baseline to 9 weeks and 6‐month follow‐up with imputed data (Expectation‐Maximization)

Measures	ICBT‐I *N* = 24 *M* (*SD*)		Control Group *N* = 24 *M* (*SD*)		Mean between‐group treatment differences (95% CI)	*p* ^d^	*d* ^e^	ICBT‐I *M* (*SD*)		Control Group *M* (*SD*)		Mean between‐group treatment differences (95% CI)	*p* ^d^	*d* ^e^
	Baseline	9 weeks	Baseline	9 weeks				Baseline	6 months	Baseline	6 months			
ISI^a^	15.92 (3.878)	11.64 (4.679)	16.29 (4.112)	14.85 (4.057)	−2.9 (−4.9 to −0.9)	.004	0.73	15.92 (3.878)	11.03 (4.814)	16.29 (4.112)	13.34 (5.024)	−2.1 (−4.7 to 0.5)	.111	0.47
SF‐12 PCS^b^	–	–	–	–	–	–	–	40.46 (8.273)	44.52 (10.439)	39.71 (10.003)	40.44 (10.178)	3.5 (−1.2 to 8.2)	.137	0.40
SF‐12 MCS^c^	–	–	–	–	–	–	–	50.07 (11.680)	51.76 (9.364)	46.30 (11.893)	46.90 (9.948)	3.4 (−1.7 to 8.5)	.182	0.50

^a^ Insomnia Severity Index.

^b^ 12 Item Short Form Survey, Physical Component Score.

^c^ 12 Item Short Form Survey, Mental Component Score.

^d^ *p*‐value.

^e^ Cohen's *d*.

### 4.3 Secondary outcomes

The mean score for SF‐12 PCS improved in the I‐CBTI group (40.46 at baseline versus 44.52 at the 6‐month follow‐up) and control group (39.71 at baseline vs. 40.44 at the 6‐month follow‐up), but the between‐group difference did not differ significantly (3.5 between‐group mean difference, 95% CI −1.2 to 8.2, *p* = .137, Cohen's *d* = 0.40) (Table 3). Nor did the between‐group difference for SF‐12 MCS show a significant difference (3.4 between‐group mean difference, 95% CI −1.7 to 8.5, *p* = .182, Cohen's *d* = 0.50) (Table 3). Unimputed data (Appendix 1) showed a small but non‐significant increase in SF‐12 PCS mean score in the I‐CBTI group from baseline (40.46) to 6 months (44.40).

**FIGURE 2:**
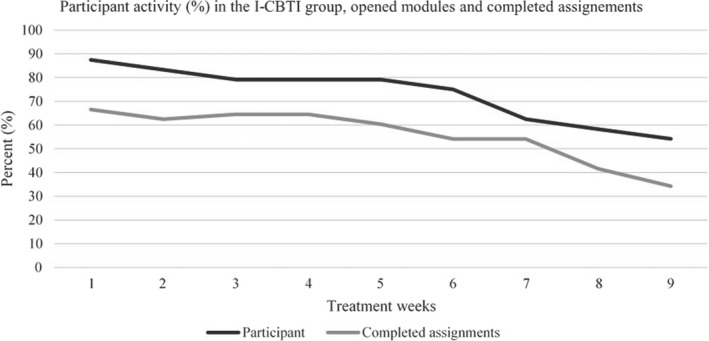
The number of active participants/module (%) and the total sum of completed assignments per module (%) in the I‐CBT group (*N* = 24). A maximum of 21 assignments were distributed in nine modules over 9 weeks

Regarding adherence in the I‐CBTI group (*N* = 24), the distribution of active participants and completed assignments/modules is presented in Figure 2. In the I‐CBTI group, 71% opened six out of nine modules and completed at least one assignment in three to six modules, and about 50% opened nine modules and completed assignments in at least seven modules. The completion of assignments was highest in the first five modules (67%), with an isolated decline in module 2 and then gradually decreasing to its lowest level of completion in module 9 (Figure 2). One participant (4%) was not active with assignments but reported reading all the text in the modules. In the control group, 16 participants (67%) opened all three modules. Health concerns, limited computer skills, other life priorities, difficulties adapting to the program or lack of motivation were reasons for non‐adherence or drop‐out mentioned by the participants from the I‐CBTI group to the nurse support through the communication system in the Iterapi platform.

**FIGURE 3:**
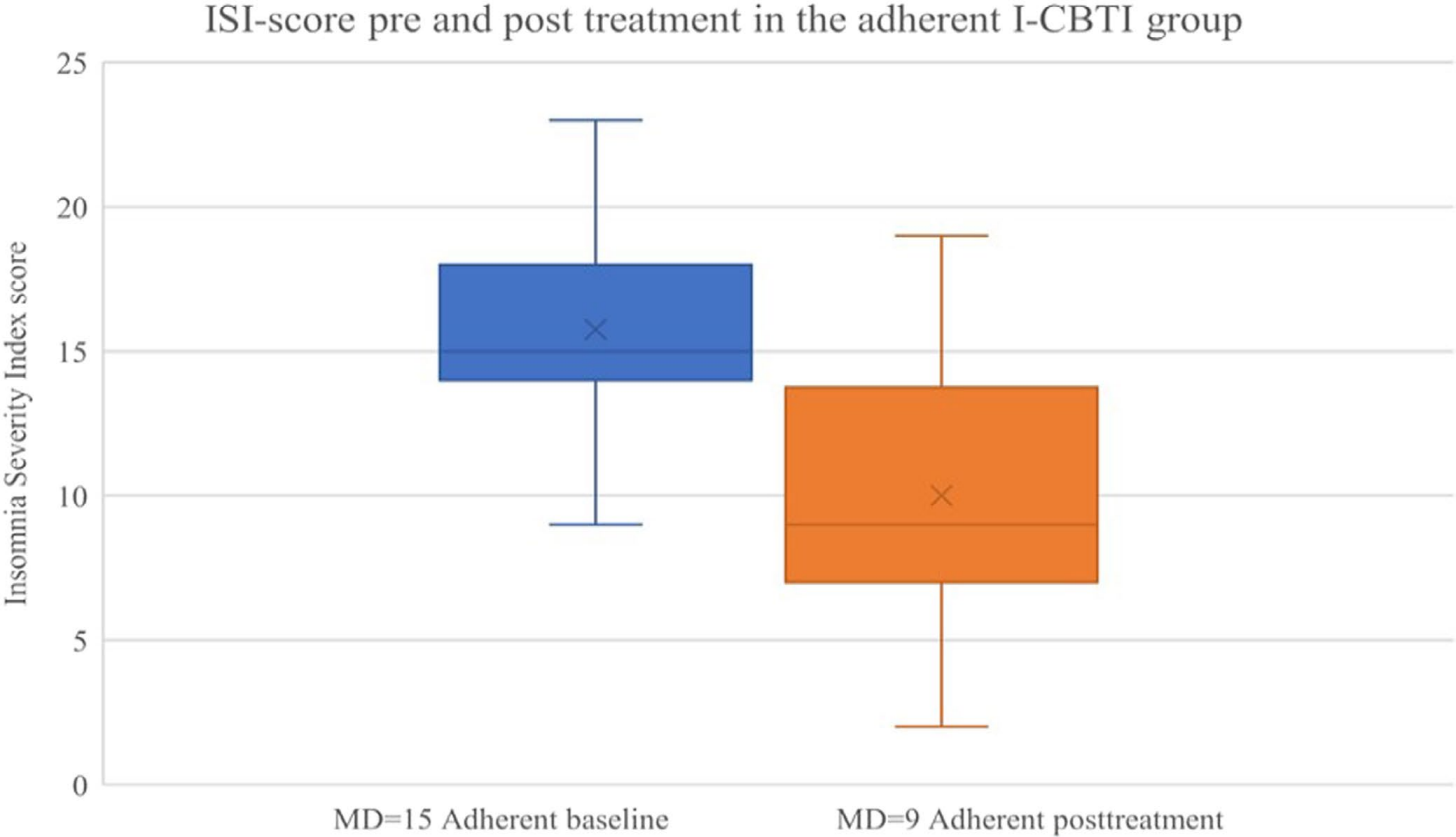
The adherent I‐CBTI group individual ISI score from baseline to post‐treatment. Adherent participants (*N* = 16) completed ≥ 5 modules in the program. Two participants (ISI baseline value 14 and 15) who were adherent to the intervention did not complete the post‐treatment (9 weeks) measurements

**FIGURE 4:**
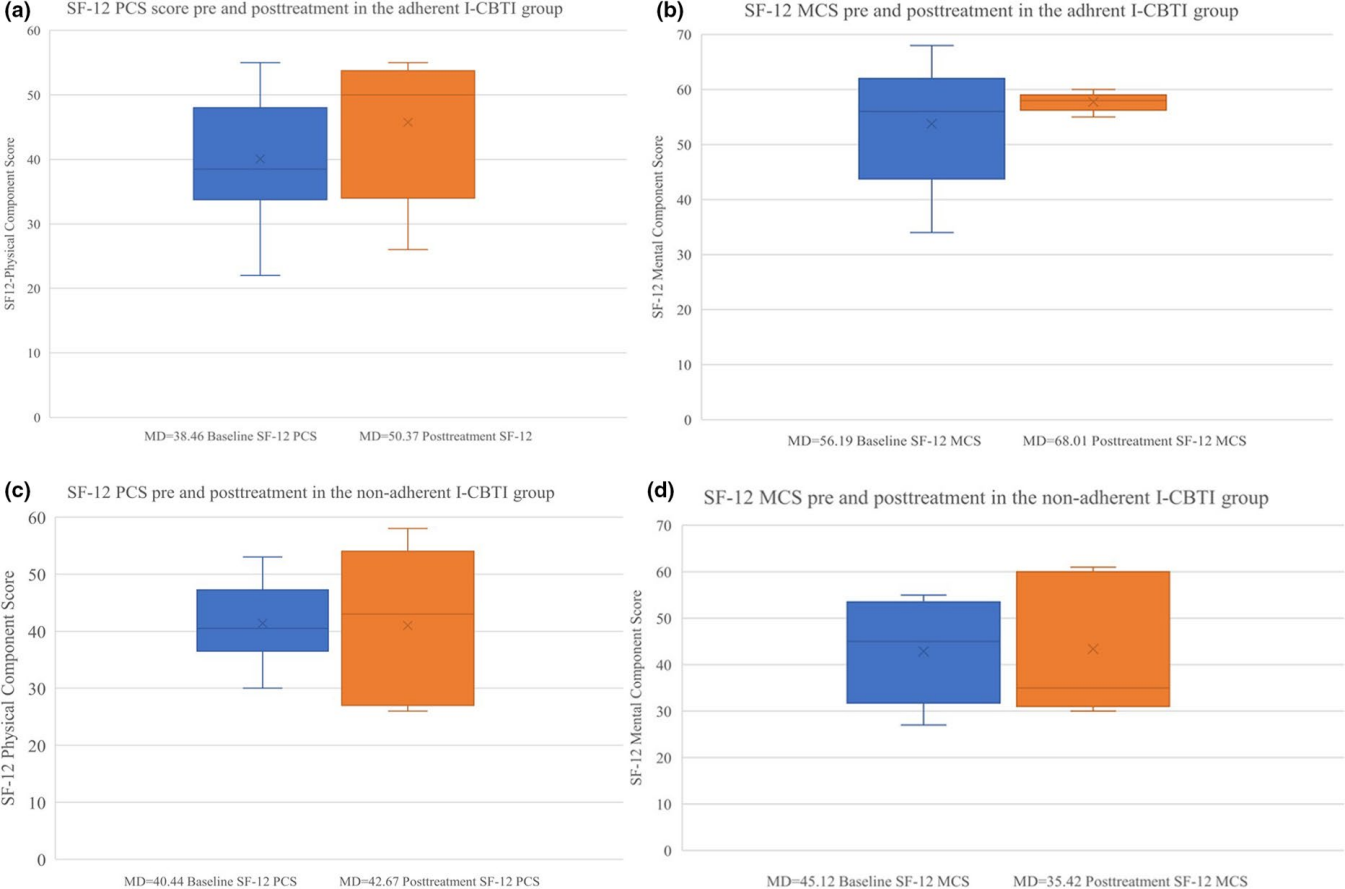
(a) The adherent I‐CBTI group (*N* = 16) SF‐12 physical component score (PCS) from baseline to follow‐up measurements (6 months). Four participants with baseline value 33, 36, 49 and 54, who were adherent to the intervention did not complete the follow‐up measurement. (b) The adherent I‐CBTI group (*N* = 16) SF‐12 mental component score (MCS) from baseline to follow‐up measurements (6 months). Four participants with baseline value 39, 42, 56 and 65, who were adherent to the intervention did not complete the follow‐up measurement. (c) The non‐adherent of the I‐CBTI group (*N* = 8) SF‐12 physical component score (PCS) from baseline to follow‐up measurements (6 months). Three participants with baseline value 38, 43 and 53, did not complete the follow‐up measurement. (d) The non‐adherent of the I‐CBTI group (*N* = 8) SF‐12 mental component score (MCS) from baseline to follow‐up measurements (6 months). Three participants with baseline value 27, 34 and 42 did not complete the follow‐up measurement

The effect on ISI and SF‐12 among adherent users is presented in Figure 3 and Figure 4a–c. The adherent group showed a significant decrease in median ISI score from baseline to post‐treatment (9 weeks) (15 vs. 9, *z* = −3.082, *p* = .002); this was not found in the non‐adherent group (15.5 vs. 16, *z* = −0.577, *p* = .564). In the adherent group, there was a within‐group significant change in median SF‐12 PCS score from baseline to 6 months (38.46 vs. 50.37, *z* = −2.118, *p* = .034), whereas no significant change was seen in SF‐12 MCS (56.19 vs. 58.01, *z* = −0.628, *p* = .530). No within‐group differences were found either in SF‐12 PCS (40.44 vs. 42.67, *z* = −0.405, *p* = .686) or SF‐12 MCS scores (45.12 vs. 35.42, *z* = −0.405, *p* = .686) in the non‐adherent group.

## 5 DISCUSSION

This is, to our knowledge, the first randomized controlled trial of nurse‐led I‐CBTI on patients with CVD using a long‐term follow‐up. We found significant improvements in insomnia symptoms (primary outcomes) in the intervention group compared to the control group, and these appeared to last over time.

This trial was developed specifically to treat insomnia in patients with CVD and used customized modules focusing on psychoeducation in pathophysiology, treatment, and self‐care of CVD (i.e. in module 2) and impact of heart disease on sleep (i.e. in module 4), as well as insomnia. The mean between‐group differences in the intention to treat analyses for ISI score from baseline to 9‐week follow‐up (post‐treatment), −2.9 between‐group mean difference, 95% CI −4.9 to −0.9 are, according to the clinical global improvement assessment, considered to be a slight ISI‐score improvement (Morin et al., 2011). It is notable that the Cohen's *d* effect size was 0.73, almost reaching the level for a large effect. Our result is partly comparable to the RCT of Lancee et al. (2016) that compared a 6‐week online CBTI treatment group with face‐to‐face CBTI, as well as a wait‐list group (post‐treatment Cohen's *d* effect of 1.0 between online CBTI and wait‐list) in terms of insomnia treatment effect. The coaches provided feedback by email based on exercises, forms and a sleep diary. Moreover, their trial had a similar group sample size (approximately 30 participants in each group) and completion rate (50% completed all six sessions), but it was based on a sample derived from the general population with no participants with CVD. The results from the current study are also difficult to compare with other I‐CBTI studies due to the other studies' dissimilar methods, such as having no active control group (Luyster et al., 2020), making comparisons between online and face‐to‐face treatments (Blom et al., 2015; Lancee et al., 2016), a younger mean age, larger sample sizes, and inclusion of participants from the general population (Kaldo et al., 2015; Lancee et al., 2016; van Straten et al., 2014) with different comorbidities, in many cases chronic conditions (Mehta et al., 2019) or participants with mental health disorders (Hagatun et al., 2018). Importantly, no previous I‐CBTI intervention with an RCT design or a long‐term follow‐up has been conducted among patients with CVD. Therefore, the significant improvement in ISI score (Cohen's *d* effect size of 0.73) achieved in the current study should be considered a successful outcome since it is a newly developed intervention format for patients with CVD and insomnia and there was an active control group.

There was a small but non‐significant improvement in both SF‐12 PCS and MCS mean scores in the unimputed data from baseline to the 6‐month follow‐up. Quality of life can be a complex matter to measure among patients with comorbid conditions, and improvements in function might be measurable long after the insomnia improvements stabilize (Kyle et al., 2010). Six months' follow‐up in combination with a small sample size could contribute to the non‐significant findings in the current study. Therefore, the number of participants is an important factor when interpreting the results. Including patients with CVD in an I‐CBTI study can be challenging since this study population includes older persons (North & Sinclair, 2012), whose use of Internet devices varies, depending on their health status (i.e. poor near vision and/or frailty) and socioeconomic level (Keranen et al., 2017).

Adherence to the I‐CBTI intervention was good during the study, but there was a 12.5% decline in adherent participants (i.e. 75%–62.5%) after module six, which focused on sleep restriction. Sleep restriction is effective for improving insomnia but can be experienced as a stressor through pressure to sleep within a restricted time period, and concerns about consequences the next day (Kyle et al., 2011). Therefore, the choice to initially limit sleep restriction to once or twice per week on account of the participants' comorbid condition, instead of giving a traditional insomnia treatment with a more rigorous sleep restriction requirement (Blom et al., 2015; Kaldo et al., 2015), was probably beneficial for preventing higher non‐adherence in the I‐CBTI group. This sleep restriction limitation, however, could have reduced the effect on the outcomes in the adherent I‐CBTI group. The reasons for drop out from I‐CBTI studies are seldom fully investigated and may vary. However, via communication with the nurse support through the Iterapi platform, participants in the current study gave indications of health strains, limited computer skills, other life priorities, difficulties adapting to the programme or lack of motivation as reasons for non‐adherence and/or intervention drop out. Difficulties logging in to the I‐CBTI or self‐study programme were mentioned as reasons for non‐adherence, and these could be explained by the study group's higher mean age (72), since computer literacy varies in the older population (Keranen et al., 2017). In an I‐CBTI intervention, Yeung et al. (2015) identified longer total sleep time, greater level of depression and lower level of ISI score as predictors for study drop out. Blom et al. (2016) presented facilitating and hindering factors for working with I‐CBTI or I‐CBT for depression. Facilitating factors were to accept having sleeping problem, negative thoughts and cognition, and barriers for working with I‐CBTI or I‐CBT were to have depression or other comorbidities. The adherent group in the current study showed a within‐group significant improvement of insomnia severity and physical quality of life but showed no significant improvement in mental quality of life (Figure 4a,b). The latter might be explained by an already high level of mental quality of life at baseline (56.19). Therefore, high mental quality of life, along with poor physical quality of life, could be motivating factors for adherence to I‐CBTI among patients with CVD. On the other hand, low mental quality of life could be associated with low levels of initiative and motivation for behavioural change. The level of symptom severity of a CVD diagnosis can result in various mental and physical challenges, influencing treatment adherence. Therefore, future research should evaluate treatment suitability from physical and psychological outcomes association with treatment adherence among patients with CVD participating in an I‐CBTI intervention.

In the current study, the I‐CBTI intervention included modules with CVD‐specific educational material. McCombie et al. (2015) suggest that interventions might be more effective and engaging if they were disease‐specific, so the patients could relate to the content of the intervention. The current study used nurse support, which was also applied in an earlier customized randomized I‐CBT study to treat depression in patients with CVD (Johansson et al., 2019) with positive results on depression. Therapist‐guided support has been shown to improve adherence to treatment protocols in I‐CBT interventions compared to self‐guided I‐CBT (Mehta et al., 2019). Moreover, previous research has suggested that the qualification requirements to deliver I‐CBT are of minor importance, although related to the dose of support‐responses (Baumeister et al., 2014). The registered nurse in the present study gave appreciative, confirmatory, advisory and reflective support to motivate the participants to complete the I‐CBTI programme. The support was delivered on‐demand, not given at certain time points, and followed the participants' needs and progress through the intervention, which has been proven to be an effective approach in other studies (Baumeister et al., 2014). Importantly, with the use of this nurse support, we obtained significant improvements on the primary outcome (i.e. insomnia symptoms). However, with full access to a detailed medical history, as is common in clinical settings, an even more tailored and patient‐centred (i.e. with focus on CVD‐specific symptoms causing insomnia problems) nurse support could be used. The nurse support in the current study composed individual feedback for each participant which could be considered as time consuming in clinical practice. Adjustments could be that some of the feedback may be standardized or only given on certain days of the week. To fit clinical practice, these adjustments should be further evaluated in future research.

Social support is an important mediator for behavioural change if the patient has a clear outcome expectation and risk perception, both of which can be motivators to enhance self‐efficacy to achieve behavioural change (Schwarzer, 2008). Moreover, secondary outcomes, such as self‐efficacy, could be important mediators to achieve long‐term management skills in patients with chronic disease (Mehta et al., 2019). It is common in behavioural change interventions that persons do not behave in accordance with their planned intention. Unexpected situations occur and people succumb to temptations. It is therefore essential to promote self‐efficacy and help the patients to convert intentions into behavioural change actions (Schwarzer, 2008). To understand the facilitating mediators for behavioural change in I‐CBTI tailored for patients with CVD and insomnia, further research is needed to explore the patient's experience, attitudes and belief towards the feasibility of such interventions. Future studies could use both qualitative and quantitative research approaches to explore facilitating and hindering factors to develop and maintain patient's self‐efficacy for behavioural change when using a tailored I‐CBTI intervention. This nurse‐led I‐CBTI study has shown significant improvement in insomnia outcomes. Future research on nurse‐led I‐CBTI research for patients with CVD would require greater power to confirm both insomnia and quality of life outcome improvements.

### 5.1 Limitations

ISI is a well‐validated instrument to detect self‐reported insomnia but should be complemented with clinical examination to confirm the insomnia diagnosis (Riemann et al., 2017). We used subthreshold insomnia symptoms according to ISI (ISI score 0 – 7 = absence of insomnia) to identify participants before they met a sleep physician. A lower cut‐off level is often used in research to identify insomnia in the general population, whereas an ISI score cut‐off at >10 or 11 would have been an alternative for optimal sensitivity of insomnia detection in a population like ours (Morin et al., 2011). However, the inclusion process in this study has strong methodological validity in terms of confirming the insomnia diagnosis of the participants by an experienced sleep physician and detecting and excluding patients with other sleep conditions not suitable for I‐CBTI treatment (i.e. sleep apnoea). A limitation of the study is the lack of information regarding time interval of the participants CVD diagnosis and specific information of disease severity, since it might have been factors influencing the outcome. Another limitation is that the requirement to include at least 60 participants (30/group) was not achieved. However, the fact that there were no significant baseline differences between the intervention and control groups in our RCT design was a strength which decreased the risk of selection bias. Furthermore, the use of an active control group provides evidence that the significant primary outcomes are probably due to the I‐CBTI content rather than increased activity or awareness of the situation (Mehta et al., 2019). Moreover, even if the use of sleep medication in the intervention and control groups relied on self‐reports, which is a limitation, the distribution of prescribed sleep medication was equal in both groups. The missing data are explained to be missing at random from the participants' point of view and is statistically supported by a non‐significant MCAR test. EM was used as an imputation approach to replace missing data to allow ITT analysis to be performed. This approach may be a limitation of the study due to its single imputation procedure.

## 6 CONCLUSION

This is probably the first study that has tested the effect of a nurse‐led I‐CBTI intervention tailored for patients with CVD and insomnia and that has shown improvement in insomnia outcomes lasting over time. These findings also suggest that nurses should be considered for delivering support in I‐CBTI interventions to increase treatment access in cardiovascular care. Future studies with a larger sample are needed to confirm these findings, complemented with CVD‐specific outcomes. Nevertheless, these findings are a starting point to develop a more individual and suitable I‐CBTI intervention for patients with CVD and insomnia.

## CONFLICTS OF INTEREST

The authors have no financial or personal conflicts to declare in regard to this study.

## AUTHOR CONTRIBUTIONS

SS, AB, MU and PJ: Intervention and study design. SS and MU: Data collection and analysis. SS, AB, PJ, MU, LJ and GA: Manuscript preparations.

## Supporting information

Supplementary Material

## Data Availability

The data are not publicly available due to ethical restrictions. The data that support the findings of this study can be available from the corresponding author upon reasonable request.

## APPENDIX 1

Treatment effect on primary and secondary outcomes from baseline to 9 weeks and 6‐month follow‐up calculated on unimputed data.

**Table:**

Measures	ICBT‐I *M* (*SD*)		Control Group *M* (*SD*)		Mean between‐group treatment differences (95% CI)	*p* ^d^	*d* ^e^	ICBT‐I *M* (*SD*)		Control Group *M* (*SD*)		Mean between‐group treatment differences (95% CI)	*p* ^d^	*d* ^e^
	Baseline *N* = 24	9 weeks *N* = 18	Baseline *N* = 24	9 weeks *N* = 20				Baseline *N* = 24	6 months *N* = 17	Baseline *N* = 24	6 months *N* = 21			
ISI^a^	15.92 (3.878)	11.06 (4.988)	16.29 (4.112)	14.70 (4.231)	−3.3 (−5.7 to −0.8)	.011	0.79	15.92 (3.878)	10.53 (5.591)	16.29 (4.112)	13.57 (5.335)	−2.6 (−5.9 to 0.8)	.127	0.56
SF‐12 PCS^b^	–	–	–	–	–	–	–	40.46 (8.273)	44.40 (11.704)	39.71 (10.003)	40.60 (10.891)	4.6 (−1.4 to 10.5)	.128	0.34
SF‐12 MCS^c^	–	–	–	–	–	–	–	50.07 (11.680)	53.51 (10.233)	46.30 (11.893)	46.97 (10.652)	4,5 (−2.1 to 11.2)	.175	0.63

^a^ Insomnia Severity Index.

^b^ 12 Item Short Form Survey, Physical Component Score.

^c^ 12 Item Short Form Survey, Mental Component Score.

^d^ *p*‐value.

^e^ Cohen's *d*.
